# Note from the editors: As time goes by

**DOI:** 10.2807/1560-7917.ES.2017.23.2.180111-1

**Published:** 2018-01-11

**Authors:** Eurosurveillance editorial team

**Affiliations:** 1 European Centre for Disease Prevention and Control (ECDC), Stockholm, Sweden

Fuelled by technologies that allow new ways of generating, disseminating and accessing scientific information, science communication inevitably undergoes changes. Scientists, publishers and editors drive or follow the evolution of bioinformatics, pre-print repositories, open access, open data, social media and bookmarking and many other things for the benefit of creating, sharing and disseminating knowledge. As time goes by, also the *Eurosurveillance* editors have not been sitting still, and 2017 brought several important changes for the journal.

First and foremost, we said goodbye to our static old website and launched a new website to adapt to changes in the publishing business and expectations of readers and contributors [1]. The new website offers interactive features, allows saving of customised searches, sharing articles on social media, social bookmarking and tracing the fate of articles via citation alerts and various article-level metrics. Authors are able to monitor page views, PDF downloads and who cited their article. A list of social media activities for individual articles can be accessed through the Altmetric doughnut.

In 2017, three associate editors and several national advisors ended their tenures. Dr Andrea Ammon, the newly elected Director of the European Centre for Disease Prevention and Control (ECDC) [2], was one of them. She left the editorial board to mark the editorial independence of the journal from its publisher, the ECDC, and reassured the Editor-in-chief of her continued support for the journal and her commitment to its editorial independence also in the future. We are grateful for this and for the funding received by ECDC in the past and in the years to come. The editors are also very thankful to all other past and present board members. We highly appreciate their constructive and wise advice and moral support.

As part of constant improvements and evolution, we changed some of our instructions for authors and editorial policies. We introduced structured abstracts for research and review articles, refined instructions for Rapid communications and amended our policy on overlapping publication to be even more in tune with the International Committee of Medical Journal editors (ICMJE) recommendations. Submissions received after the implementation of these changes evidenced that many of our authors have picked them up fast and this lets us believe that our new instructions for outbreak reports will be noted fast as well. The new policy on supplementary materials and the policy for making genomic sequences publically available should contribute to even greater transparency, reproducibility and reuse of data.

While there were many changes, the interest in our journal has not changed over the past year and our indices continued to be favourable. We received over 900 submissions (266 Rapid communications, 606 regular articles and 29 ‘other’) from across the globe. Some 500 experts reviewed for us and helped us select 187 scientific papers for publication (69 Rapid communications, 118 regular articles). In addition, we published eight editorials, 11 letters and 24 miscellaneous items. Our 2017 acceptance rate of 21% (Rapid communications 26%, regular articles 19%) was similar to that in the previous two years. The decision to process and publish or not was not always an easy one. However, with the help of our associate editors, advisors and peer-reviewers we believe we selected interesting, good quality and public health-relevant articles. And as every year, we are obliged to all our supporters and contributors who help us shape the journal’s content and who have an open ear for us whenever we need them. We are also grateful to colleagues at ECDC and other close supporters who are there for us and who act as ambassadors for the journal.

With a figure of 7.2, the impact factor released in mid-2017 was the highest ever for *Eurosurveillance.* The journal continued to rank among the top 10 in its category in the Journal Citation reports for the sixth year in a row. The journal also remained in the first quartile (for all categories listed) in the Scimago journal rank and Google Scholar metrics were also favourable. The newly introduced Sopus-based CiteScore for *Eurosurveillance* is 3.7 (96th CiteScore Percentile), which corresponds to rank 15 among 449 journals in the category Medicine: Public Health, Environmental and Occupational Health.

As in previous years, we continued to cover outbreaks and relevant public health events in a timely manner. An emerging topic covered in several papers in 2017 was the hepatitis A outbreaks among men who have sex with men and the journal featured several articles with new findings on plasmid-encoded colistin resistance. Influenza, HIV/AIDS and food-borne diseases were other topics in several *Eurosurveillance *issues. Acknowledging the importance of vaccination and supporting efforts to improve documentation and coverage, we published a special issue on Immunisation Information Systems in April [3].

What can readers and contributors expect from us in 2018? As always we will strive to provide interesting, scientifically sound and public health-relevant articles and timely information for public health action. We will intensify our presence on social media and have started to be more proactive using LinkedIn as a further channel to disseminate information. As a result from a call for papers published in March 2017, we will publish a special issue on screening and prevention of communicable diseases in newly arrived migrants in Europe in the coming months and we are looking forward to receiving submissions for an ongoing Call for papers for a special issue on advanced diagnostics to inform public health policy [4].

The new website features enables the publication of educational materials in the form of structured questions and quizzes and supplements to go along with articles. We plan to launch an educational series later in the year, so stay tuned !

The year 2018 marks the 200th anniversary of Ignaz Semmelweis’ birth, a pioneer of public health and infection control. His conclusions that hand washing could interrupt the transmission of infections in the hospital setting were based on his observation of marked differences between two clinics in Vienna in the mid-18th century with regard to the occurrence and fatal outcome of puerperal fever in mothers or their babies. After successful implementation of hand washing for doctors, the morbidity and mortality in the maternity ward where he worked dropped considerably [5]. However, his ideas challenged the established scientific and medical opinions and were refused by the medical community. Semmelweis tragically died at the age of 47 years. The significance and validity of his findings were only acknowledged after his death. They still continue to be important and valid today. To honour Semmelweis, *Eurosurveillance* will highlight articles on hospital-associated infections and their control published in 2018 in a special collection ‘Hospital infection control – 200 years after the birth of Ignaz Semmelweiss’.

We look forward to an interesting year with contributions on new findings and opportunities that help prevent and control infectious diseases in Europe and beyond.

## References

[1] Eurosurveillance editorial team. Note from the editors: Wave goodbye and say hello - Eurosurveillance to launch new website soon. Euro Surveill. 2017;22(38):30617. 10.2807/1560-7917.ES.2017.22.38.30617 PMC5709947 28935024

[2] Eurosurveillance editorial team Eurosurveillance editorial team. Third Director of the European Centre for Disease Prevention and Control takes office. Euro Surveill. 2017;22(25):30560. 10.2807/1560-7917.ES.2017.22.25.30560 28662765 PMC5490457

[3] Special edition: Immunisation Information Systems. Euro Surveill. 2017;22(17). Available from: https://www.eurosurveillance.org/upload/site-assets/imgs/Special_Issue_Immunisation_print.pdf 10.2807/1560-7917.ES.2017.22.17.30519 PMC5434883 28488999

[4] Eurosurveillance editorial team. Call for papers for a special issue on advanced diagnostics to inform public health policy. Euro Surveill. 2017;22(41):171012-1.

[5] lgnaz Semmelweis. The etiology, concept, and prophylaxis of childbed fever. Trans. K. Codell Carter. Madison: The University of Wisconsin Press; 1983.

